# The Surgical Odyssey: A Case Report on Rare Isolated Abdominal Wall Recurrence in an Adult Ovarian Granulosa Cell Tumor

**DOI:** 10.7759/cureus.74708

**Published:** 2024-11-28

**Authors:** Monal Garg, Ritu Ahlawat, Jimmy Mathew, Priya Bhati, Sam Thomas, Sanju Samuel

**Affiliations:** 1 Gynecologic Oncology, Amrita Institute of Medical Sciences, Kochi, IND; 2 Plastic and Reconstructive Surgery, Amrita Institute of Medical Sciences, Kochi, IND

**Keywords:** abdominal reconstruction, abdominal wall surgery, adult-type granulosa cell tumor, neoplasm recurrence, rectus muscle flap

## Abstract

A 50-year-old woman with a history of adult granulosa cell tumor (AGCT) of the right ovary was under follow-up after undergoing several surgeries, including a total abdominal hysterectomy with bilateral salpingo-oophorectomy. She was initially diagnosed eight years ago and remained disease-free for 52 months. However, she later experienced a recurrence, indicated by elevated inhibin B levels (58 ng/mL) and the presence of peritoneal soft tissue tumors. Following secondary debulking surgery, histopathology confirmed recurrent granulosa cell tumor (GCT). Although she was advised to undergo hormonal therapy, she was lost to follow-up. After 21 months, she returned with an increased inhibin B level of 82 ng/mL, but imaging showed no signs of recurrence. Following a disease-free interval (DFI) of 23 months, an abdominal lump was discovered, prompting tertiary debulking surgery. During this procedure, cystic lesions were excised, and a large defect in the rectus sheath was repaired using a left rectus abdominis flap, followed by the placement of a mesh. Postsurgery, she received adjuvant chemotherapy consisting of paclitaxel and carboplatin. She had a favorable recovery with no complications during the postoperative period. At the six-month follow-up, her wound had healed well, and she was doing well overall. Isolated recurrent AGCT on the abdominal wall is a very rare presentation and can be managed using debulking surgery, with a multidisciplinary team playing a crucial role.

## Introduction

Granulosa cell tumor (GCT) is a relatively uncommon malignant tumor originating from the sex cord-stromal cells and accounts for only 2%-5% of all ovarian carcinomas. Clinical characteristics and histological features divide GCT into adult GCT (AGCT) and juvenile GCT (JGCT). AGCT differs from JGCT as it is much more common, making up about 95% of all GCTs. AGCT can occur in women across ages, but it is typically detected between 50 and 54 years [1]. About 22.5% of patients have no noticeable symptoms when diagnosed. It is often identified at an early stage and generally has a favorable prognosis compared to patients with other types of ovarian cancers. Surgery is a critical part of both initial and postrelapse treatments for GCT. Adjuvant therapy, however, is still being explored and requires further research. GCT recurrence is rare, typically occurring between five and 30 years after the initial diagnosis. Predicting recurrence is challenging, and factors such as tumor size, stage, and mitotic index are frequently linked to recurrence. Furthermore, the location of recurrence may vary depending on the clinical stage or surgical procedures [2].

## Case presentation

**Figure 1:**
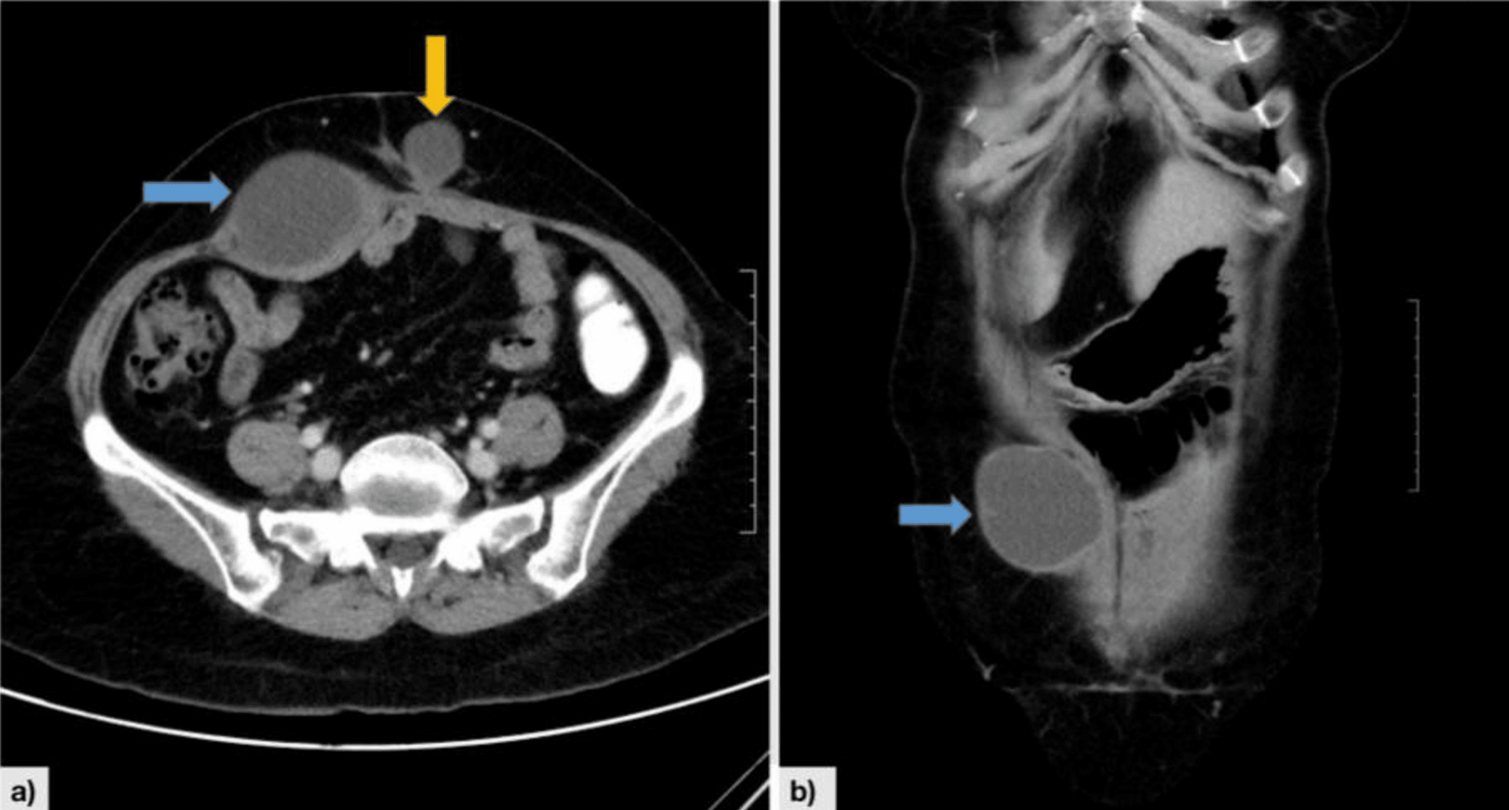
This contrast-enhanced computed tomography of the abdomen and pelvis image shows recurrent lesions (second time) in the form of two hypodense cystic lesions in the axial (a) and coronal (b) sections. One is located in the anterior abdominal wall in the right rectus abdominis muscle, measuring 5.3 x 7.3 cm and indicated by a blue arrow. The other lesion is in the subcutaneous plane along the left paramedian aspect of the midline, measuring 2.5 x 2.8 cm and indicated by a yellow arrow.

We present a case of a 50-year-old, uniparous lady who was diagnosed with AGCT of the right ovary and came for a routine follow-up. She was diagnosed with AGCT eight years ago after having a total abdominal hysterectomy with bilateral salpingo-opherectomy (TAH-BSO) at a different hospital for an adnexal mass. Her inhibin B levels before the surgery were not available, but after the surgery, they were normal (1.74 ng/mL). She did not undergo any additional treatment or hormonal therapy. After being disease-free for 52 months, she experienced a recurrence with elevated inhibin B levels (58 ng/mL), and a contrast-enhanced computed tomography (CECT) abdomen and pelvis indicated new peritoneal soft tissue tumor deposits (3 x 3 cm) in the left iliac fossa. She underwent secondary debulking surgery, including total omentectomy with excision of peritoneal deposits in the left iliac fossa. The histopathology report suggested recurrent GCT with immunohistochemistry showing cells positive for inhibin and negative for cytokeratin (CK) and synaptophysin. Following this, she was lost to follow-up. She returned after 21 months of disease-free interval (DFI) with an increased inhibin B level of 82 ng/mL. No recurrence was depicted in the CECT abdomen and pelvis, so she was kept under a three-monthly follow-up with inhibin B levels and imaging only in case of rising inhibin B levels. Following a DFI of 23 months of biochemical recurrence, she noticed an abdominal lump, which was around 6 x 7 cm on the right side of the midline infraumbilically. On CECT abdomen and pelvis, two cystic lesions were present in the anterior abdominal wall, right rectus abdominis muscle (5.3 x 7.3 cm), and in the subcutaneous plane along the left paramedian aspect of the midline (2.5 x 2.8 cm) (Figure 1).

**Figure 2:**
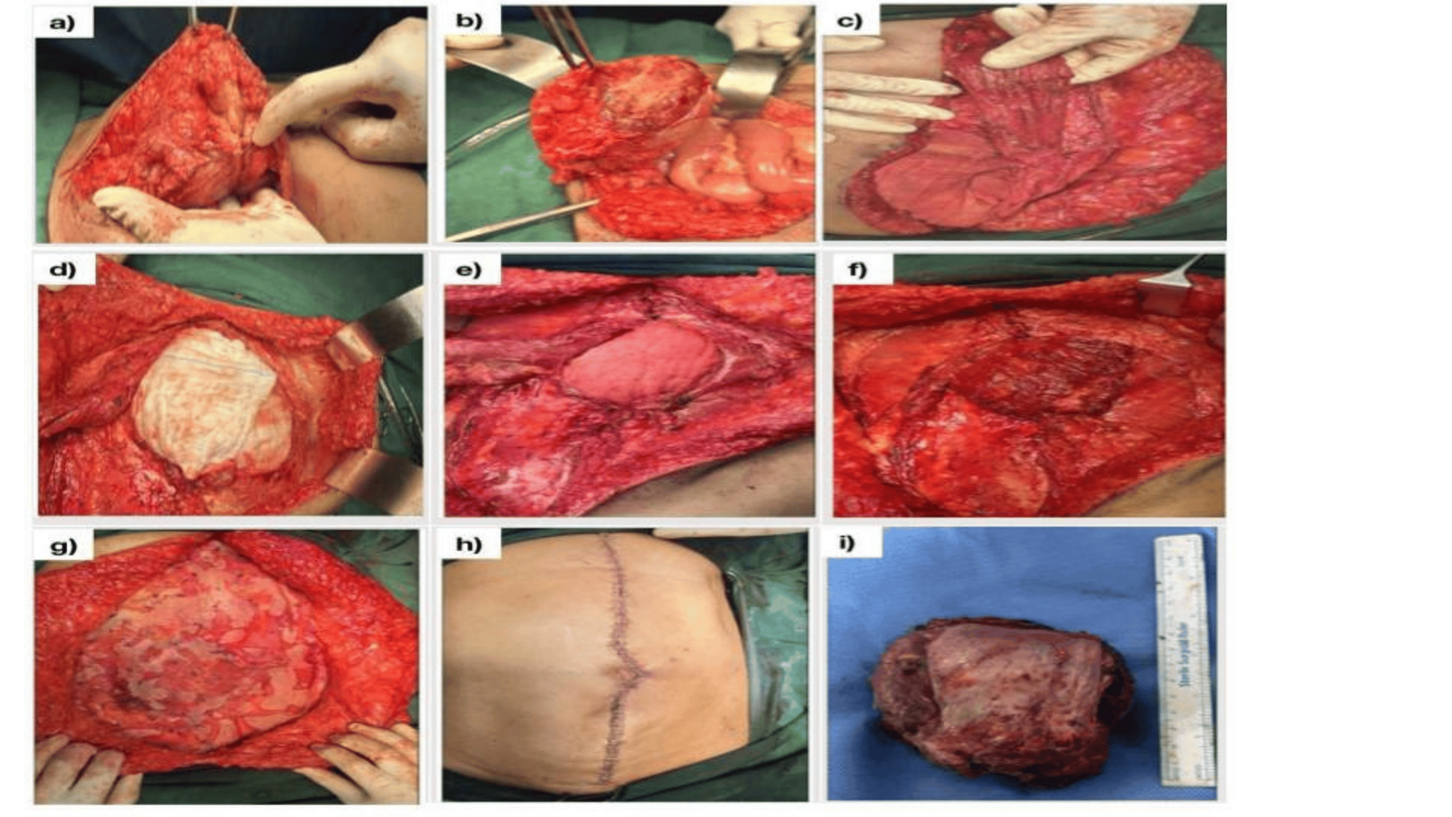
(a) Tumor in the right rectus abdominis muscle (5.3 x 7.3 cm). (b) Another tumor deposit in the subcutaneous plane along the left paramedian aspect of the midline (2.5 x 2.8 cm). (c) Part of the elevated inferiorly based left rectus abdominis muscle. (d) Abdominal wall defect showing a surgical mop covering the bowel contents. (e) Abdominal wall defect showing a surgical mop covering the bowel contents. (f) Flap inserted into the defect. (g) Prolene mesh (Ethicon, Inc., Somerville, US) placed over the abdominal wall. (h) Skin closure. (i) Resected specimen (larger tumor mass).

Following a discussion with a multidisciplinary committee during the tumor board meeting, she was scheduled for tertiary debulking surgery. Intraoperatively, a midline vertical incision was given, and the abdominal wall was separated into layers (Figure 2).

**Figure 3:**
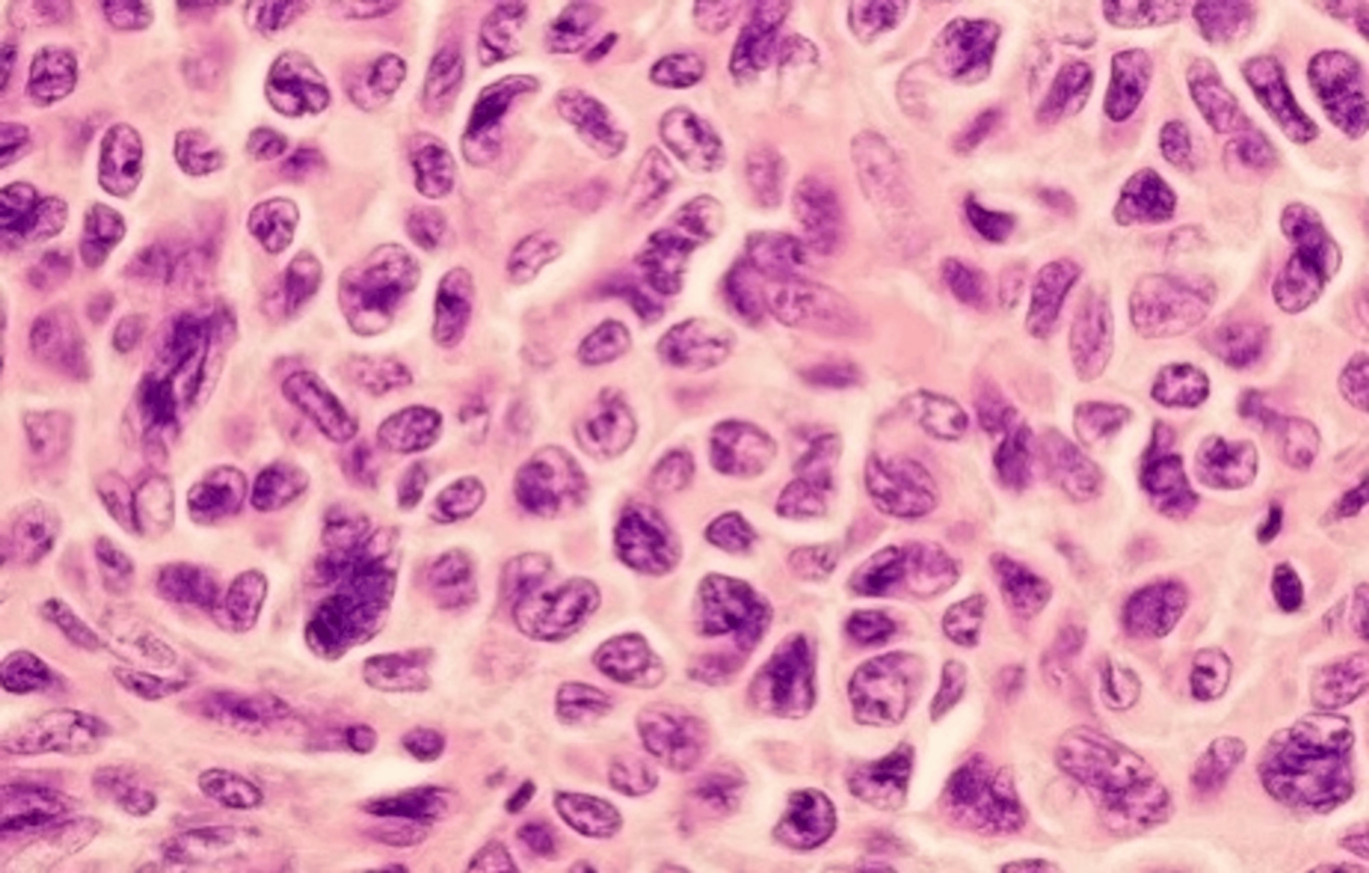
Histology picture showing gland-like structures filled with acidophilic material representing adult-type granulosa cell tumor.

The cystic lesion was excised intact, measuring 7 x 6 cm involving the subcutaneous tissue, right rectus abdominis muscle, and rectus sheath, along with another 3 x 3 cm involving the left rectus abdominis muscle and rectus sheath. Right rectus abdominis defect of size 15 x 10 cm was found inferiorly. Primary reconstruction with a left inferiorly based rectus abdominis flap with bilateral component separation was done. The left inferiorly based left rectus abdominis flap was raised and flipped over to cover the defect. The bilateral external oblique sheath was incised 2 cm lateral to the linea semilunaris. The plane between the external oblique and internal oblique was dissected and freed. This gave a movement of 2 cm. A 30 x 30-cm mesh was placed from the xiphisternum to the lower edge of the defect, and the mesh was sutured all around the external oblique aponeurosis. The wound was closed primarily in two layers. A subcutaneous drain was placed. She resumed oral diet on postoperative day one. No complication was experienced during her postoperative period, and she was discharged on the fifth day of surgery after removing the subcutaneous drain on the fourth postoperative day. The histopathology report suggested recurrent AGCT of the ovary as depicted in Figure 3.

She received six cycles of adjuvant chemotherapy (paclitaxel and carboplatin), which was started three weeks postoperatively. After six months of follow-up, her wound is well healed and she is doing well.

## Discussion

Adult ovarian GCTs (AOGCTs) are more prevalent, accounting for 95% of all GCTs [1]. They typically occur in middle-aged women and often manifest as an asymptomatic mass, abdominal pain, abnormal vaginal bleeding, and precocious puberty. They commonly present as stage I disease and have an excellent five-year survival rate of over 90% [1,2].

Surgery is essential for diagnosing and managing suspected GCTs [3]. The standard recommended procedure is TAH-BSO [3]. Lymphadenectomy is debatable due to a very low incidence of lymph node metastasis in GCTs [4]. Women who wish to maintain fertility may undergo unilateral salpingo-oophorectomy if the tumor is localized to a single ovary. Nevertheless, it is advisable to undergo complete surgical staging after completing childbearing [4]. Adjuvant systemic therapy is required for stages Ic and above, and the most commonly used systemic therapy treatment is bleomycin sulfate, etoposide phosphate, and cisplatin (BEP). Studies have compared BEP therapy's efficacy and side effects vs. taxane-based therapy [5]. According to a retrospective study by Brown et al., patients treated with taxane-based regimens for sex cord-stromal tumors (SCSTs) showed similar response rates and progression-free survival as those treated with BEP but experienced lower toxicity [6]. Additionally, the combination of bevacizumab with paclitaxel did not demonstrate significant clinical benefits [6]. The current targeted therapy for advanced ovarian cancer is bevacizumab [5]. Clinical trials are looking into the potential of bevacizumab in treating recurrent AGCTs. Some studies have shown the effectiveness of bevacizumab in GCTs [7]. It may help in managing symptoms related to new blood vessel formation, such as vascular endothelial growth factor (VEGF)-mediated ascites in ovarian cancer [7]. The overall response and clinical benefit rates of bevacizumab in recurrent GCTs are 38% and 63%, respectively [7]. Therefore, bevacizumab may play a significant role in controlling the progression of the disease [7].

Tumor markers play a crucial role in follow-up protocols. Studies have shown that elevated inhibin B levels can precede the clinical detection of recurrence by several months [8]. Currently, serum inhibin B is considered the most reliable tumor marker for AGCTs, with reported sensitivity ranging from 88% to 100% [8]. The anti-Mullerian hormone (AMH) has also been used as a marker for AGCT, showing similar sensitivity and specificity to inhibin B [8].

Factors that indicate a higher likelihood of cancer recurrence include advanced stages (Ic and higher), large tumor size (10-15 cm or more), poorly differentiated histology, and a high mitotic index [9]. Recurrences occur in about 25% of patients, most commonly between four and seven years after the initial treatment, and over 50% of these patients eventually die from the disease [9]. Therefore, close monitoring after surgery is crucial to detect any signs of relapse at an early stage [9]. These tumors have an unpredictable period before recurrence due to their slow progression, so some patients may experience relapse 30-40 years after the initial surgery [9]. The pelvis is the most common site for recurrence, but many patients are also at risk of cancer spreading beyond the pelvic area [10]. For this reason, regular pelvic examinations and monitoring of serum markers are important during follow-up for these tumors [10].

In the case of metastatic or recurrent disease, the primary treatment approach involves surgical cytoreduction with the goal of achieving no visible residual disease [11]. Complete resection of recurrent tumors provides the best chance for survival, although it can be challenging and may lead to increased morbidity [11]. Following complete surgical cytoreduction, paclitaxel/carboplatin or BEP can be administered. Other options such as radiation, chemotherapy, and hormonal therapy can be considered for cases with non-resectable disease [11]. Chemotherapy options may include docetaxel, paclitaxel, paclitaxel/ifosfamide, paclitaxel/carboplatin, and vincristine, Adriamycin, and cyclophosphamide (VAC). Hormone recurrence therapy options include aromatase inhibitors, leuprolide, and tamoxifen [11]. Single-agent bevacizumab or leuprolide is also an option for patients with recurrent GCTs. Palliative localized radiation therapy may also be beneficial [11-13]. Previous case reports on AGCT related to rare recurrent sites and their management are summarized in Table 1.

**Table 1:** Review of literature on recurrent granulosa cell tumors of the ovary and their management. F/B: followed by; Y: years; TAH: total abdominal hysterectomy; RSO: right salpingo-opherectomy; Rt: right; BEP: bleomycin sulfate, etoposide phosphate, and cisplatin; GnRH: gonadotropin-releasing hormone

Study	Study type	Recurrence interval	Recurrence site	Management
Bin Naeem et al. (2024) [1]	Case report of two patients	Multiple recurrences: Patient A: 1st: 8 Y (2008); 2nd: 2 Y (2010); 3rd: 3 Y (2013). Patient B: 1st: 3 Y (2007); 2nd: 1 Y (2008); 3rd: 5 Y (2013); 4th: 3 Y (2016)	Patient A: 1st: ovary; 2nd: lung; 3rd:lesion abutting sigmoid colon; disease progression (2020): lung lesion. Patient B: 1st: left adnexal mass; 2nd: pelvic mass; 3rd: left iliac fossa; 4th: pelvic side wall masses	Patient A: 1st: TAH + RSO; 2nd: left lower lobe lobectomy; 3rd: hormonal therapy; 4th: carboplatin + paclitaxel. Patient B: 1st: chemotherapy (carboplatin + cyclophosphamide); 2nd: resection of the mass F/B 4 cycles of BEP; 3rd: 6 cycles of carboplatin + paclitaxel F/B pelvic mass resection; 4th: 6 cycles of carboplatin + paclitaxel
Ohta et al. (2021) [2]	Case report	11 Y	Omentum	Resection of mass
Suzuki et al. (2023) [3]	Case report	18 Y	Chest wall and diaphragm	Video-assisted thoracoscopy surgery (VATS)
Dolkar et al. (2023) [5]	Case report	14 Y	Omentum	Chemotherapy (etoposide + cisplatin)
Fujita et al. (2015) [8]	Case report	25 Y	Liver	Extended right hepatic lobectomy + combined resection of diaphragm + cholecystectomy + omentectomy + RSO + hysterectomy
Karalok et al. (2015) [9]	Retrospective analysis	43.5 months (median)	5 patients: upper abdomen and pelvis; 3 patients: outside abdomen; 3 patients: upper abdomen; 1 patient: pelvis and outside abdomen; 1 patient: upper abdomen and outside abdomen	
Pai et al. (2022) [12]	Case report	Multiple recurrences: 1st: 23 Y (2013); 2nd: 4 Y (2017)	1st: lower abdomen; 2nd: right pre-sacral pelvis	1st: exploratory laparotomy (2013); 2nd: excision of mass + appendectomy, omentectomy, and extensive adhesiolysis
Teoh et al. (2010) [13]	Case report	Multiple recurrences: 1st: 12 Y (1991); 2nd: 4 Y (1996); 3rd: 1 Y (1997); 4th: 4 Y (2002)	1st: retroperitoneal; 2nd: retroperitoneal mass; 3rd: apex of vagina; 4th: liver	1st: removal of mass + right lymphadenectomy followed by pelvic radiation; 2nd: removal of mass + segmental resection of right hemidiaphragm; 3rd: GnRH agonist + tamoxifen F/B resection of mass with bowel resection and anastomosis; 4th: partial Rt liver resection

## Conclusions

In summary, AGCTs reoccur after a long disease-free period. Our patient experienced two recurrences. During her first recurrence, she had a secondary debulking surgery and was advised to undergo maintenance therapy, which she did not follow. Following this, she did not attend follow-up appointments, and 23 months after her previous surgery, she presented with another recurrence that could have been detected earlier. Therefore, it is important to have regular follow-up appointments, including inhibin B testing with or without imaging. A multidisciplinary approach can be beneficial in managing recurrences and can lead to better outcomes.
